# Water-soluble porphyrin-mediated enhanced photodynamic and chemotherapy employing doxorubicin for breast cancer

**DOI:** 10.1007/s10103-025-04466-z

**Published:** 2025-05-23

**Authors:** Aya Mokhtar, Tarek Mohamed, Ahmed O. Eigza, Mohamed E. El-Khouly

**Affiliations:** 1 Egypt-Japan University of Science and Technology, Alexandria, Egypt; 2 Ain Shams University, Cairo, Egypt; 3 Laser Institute for Research and Applications (LIRA), Beni-Suef University, Beni-Suef, Egypt

**Keywords:** Breast cancer, Photodynamic therapy, Chemotherapy, Porphyrin, Femtosecond laser

## Abstract

Breast cancer is the second most common cancer globally and the leading cause of cancer-related deaths in women. Current treatments, such as chemotherapy and surgery, often have side effects and can lead to drug resistance. Developing new treatments that specifically target cancer cells while minimizing side effects is essential. Combining traditional cancer treatments with photodynamic therapy (PDT) is a promising approach. This study evaluated the effectiveness of femtosecond laser-driven PDT using Doxorubicin (DOX) and tetrakis (1-methylpyridinium-4-yl) porphyrin (TMPyP), both individually and in combination, on MDA-MB-231 and T47D breast cancer cells. TMPyP-PDT and DOX monotherapy both exhibited dose-dependent cytotoxicity. However, combination therapy was more effective at lower DOX concentrations, potentially reducing side effects. This combination also increased reactive oxygen species (ROS) levels, inhibited angiogenesis by reducing TGF-β and VEGFA expression, and induced apoptosis by decreasing BCL-2 and increasing BAX levels compared to individual treatments. These findings suggest that combining TMPyP-mediated PDT with Doxorubicin could effectively inhibit breast cancer cell growth.

## Clinical trial number

Not applicable.

## Introduction

Breast cancer is the most common malignancy among women worldwide. Breast cancer patients constitute approximately 36% of all oncology patients. In 2018, an estimated 2.089 million women were diagnosed with breast cancer [1]. Breast cancer is classified into three molecular subtypes based on the expression of hormone receptors (estrogen receptor, progesterone receptor) and HER2: hormone receptor-positive (HR+), HER2-positive (HER2+), and triple-negative breast cancer (TNBC). HR + breast cancers account for 70–75% of invasive breast cancers, HER2 + for 15–25%, and TNBC for 15% [2]. Systemic treatment for breast cancer is tailored to the molecular subtype. HR + tumors are typically treated with endocrine therapy, HER2 + tumors with anti-HER2 therapy (e.g., trastuzumab) combined with chemotherapy, and TNBC with chemotherapy alone [3].

Doxorubicin (DOX), an anthracycline antibiotic, is widely used in the treatment of various cancers, including leukemia, breast cancer, and ovarian cancer [4]. DOX intercalates into DNA’s double helix and inhibits topoisomerase II, leading to DNA damage and cell death [5]. While effective, DOX’s clinical use is limited by severe side effects, such as cardiotoxicity, myelosuppression, and neurotoxicity [6]. Therefore, strategies to enhance DOX’s efficacy while mitigating its toxicity are needed. Combining low-dose DOX with other therapeutic modalities, such as photodynamic therapy (PDT), could be a promising approach.

Photodynamic therapy (PDT) offers a compelling cancer treatment strategy characterized by reduced side effects, absence of drug resistance, and enhanced selectivity [7]. PDT utilizes the interplay of a photosensitizer, light, and molecular oxygen, operating through two main pathways: (i) Type I PDT: Direct reactions between the excited photosensitizer and biomolecules, leading to reactive oxygen species (ROS) formation and oxidative damage. (ii) Type II PDT: Energy transfer to oxygen, generating singlet oxygen (¹O₂), which damages cellular components and initiates apoptosis [8].

**Fig. 1:**
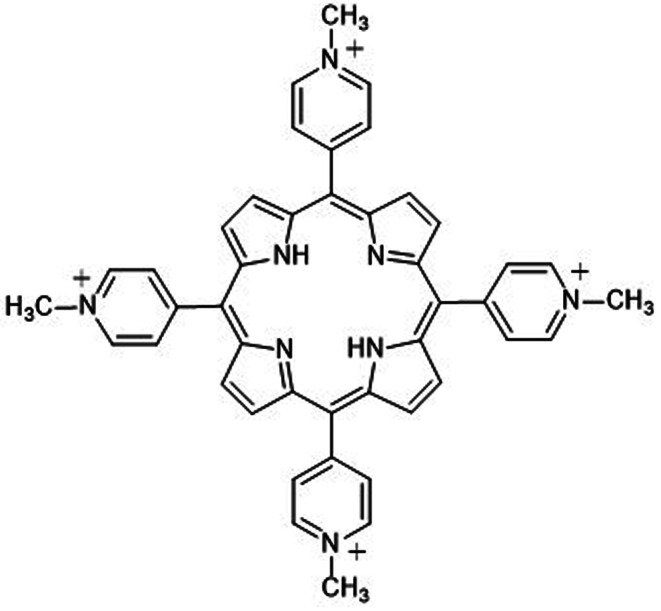
Structure of tetrakis (1-methylpyridinium-4-yl) p-toluenesulfonate porphyrin (TMPyP)

Porphyrins are commonly used photosensitizers. TMPyP (Fig. 1), a novel water-soluble porphyrin with strong electron-hole transfer ability, exhibits preferential tumor accumulation [9]. Its absorption Q bands within the 600–700 nm range align with the tissue’s optical window, facilitating light penetration [10]. Notably, wavelengths below 800 nm are essential for singlet oxygen generation, as longer wavelengths lack sufficient energy for the photodynamic reaction [11, 12].

While continuous wave (CW) lasers were traditionally used in photodynamic therapy (PDT), pulsed lasers were hypothesized to improve efficacy by enhancing tissue reoxygenation and radiation therapy [13]. Studies showed that pulsed radiation (150 J/cm²) induced greater deep tissue damage than CW lasers with first-generation photosensitizers like hematoporphyrin derivative (HpD) [14, 15]. Femtosecond pulses further increased photosensitizer photoactivation and photobleaching [16], suggesting that higher photobleaching correlates with increased singlet oxygen generation and improved PDT efficacy.

Chemo-photodynamic therapy (chemo-PDT) is an expanding research area, with in vitro studies demonstrating synergistic effects across various cancers [17]. For instance, combinations like Mce6-PDT with doxorubicin in ovarian cancer [18], ALA-PDT with mitomycin C in bladder cancer [19], and HpD-PDT with cisplatin in lymphoma [20] showed enhanced efficacy. Similar synergy was observed in lung and breast cancer with gemcitabine or cisplatin combined with HpD or indocyanine-green PDT [21, 22]. Nanoplatforms, such as gold nanorods and aptamer-functionalized nanoparticles, have been developed to deliver chemo-PDT agents (doxorubicin, TMPyP4) for targeted therapy in chemo-resistant breast cancer cells, significantly improving cell damage compared to single-drug treatments [23, 24].

This study investigated the efficacy of varying concentrations of cationic porphyrin TMPyP-mediated photodynamic therapy (PDT) using 690 nm femtosecond laser pulses to treat MDA-MB-231 and T47D breast cancer cell lines. Additionally, the cytotoxic effects of serial doxorubicin hydrochloride (Adricin) concentrations on these cell lines were assessed. Furthermore, the study explored the combined cytotoxic effects of TMPyP-induced PDT and various doxorubicin concentrations to determine optimal treatment doses. The lethal effects of TMPyP-mediated PDT, doxorubicin, and their combination were evaluated using the MTT assay to measure cell metabolic activity and by monitoring reactive oxygen species (ROS) levels. Unlike previous studies that primarily focused on macroscopic outcomes, this research aimed to investigate the molecular mechanisms underlying the effects of TMPyP-PDT, low-dose doxorubicin, and their combination on the mRNA expression of key genes involved in cancer progression: transforming growth factor-β (TGF-β), vascular endothelial growth factor-A (VEGF-A), B-cell lymphoma-2 (Bcl-2), and Bcl-2-associated X protein (Bax).

## Materials and methods

### Chemical materials

High-glucose Dulbecco’s Modified Eagle’s Medium (DMEM) and phosphate-buffered saline (PBS) were purchased from Biowest (Riverside, USA). Fetal bovine serum (FBS) and penicillin-streptomycin were obtained from Gibco (Fisher Scientific, USA). 3-(4,5-Dimethyl-2-thiazolyl)-2,5-diphenyl-2 H tetrazolium bromide (MTT) and dimethyl sulfoxide (DMSO) were acquired from Sigma-Aldrich (St. Louis, USA). α,β,χ,δ-Tetrakis(1-methylpyridinium-4-yl)porphyrin (TMPyP) was purchased from Sigma-Aldrich (St. Louis, Missouri, USA). A 2 mg/mL (1.466 mM) stock solution of TMPyP was prepared in sterile PBS (pH 7.4) and diluted to the desired concentrations in a complete growth medium before use. Doxorubicin hydrochloride (50 mg/25 mL) was obtained from Hikma Specialized Pharmaceuticals (Cairo, Egypt) and stored in the dark at 4 °C.

### Cell culture and passaging

MDA-MB-231 and T47D breast cancer cell lines were obtained from the American Type Culture Collection (ATCC, Manassas, VA, USA). Cells were thawed, resuspended in DMEM supplemented with 10% FBS and 1% penicillin/streptomycin, and cultured in T-75 flasks. Cultures were maintained in a humidified incubator at 37 °C with 5% CO_2_. The media was refreshed every 2–3 days. Cells were subcultured upon reaching 90% confluence.

### Optimization and setup of laser systems

**Fig. 2:**
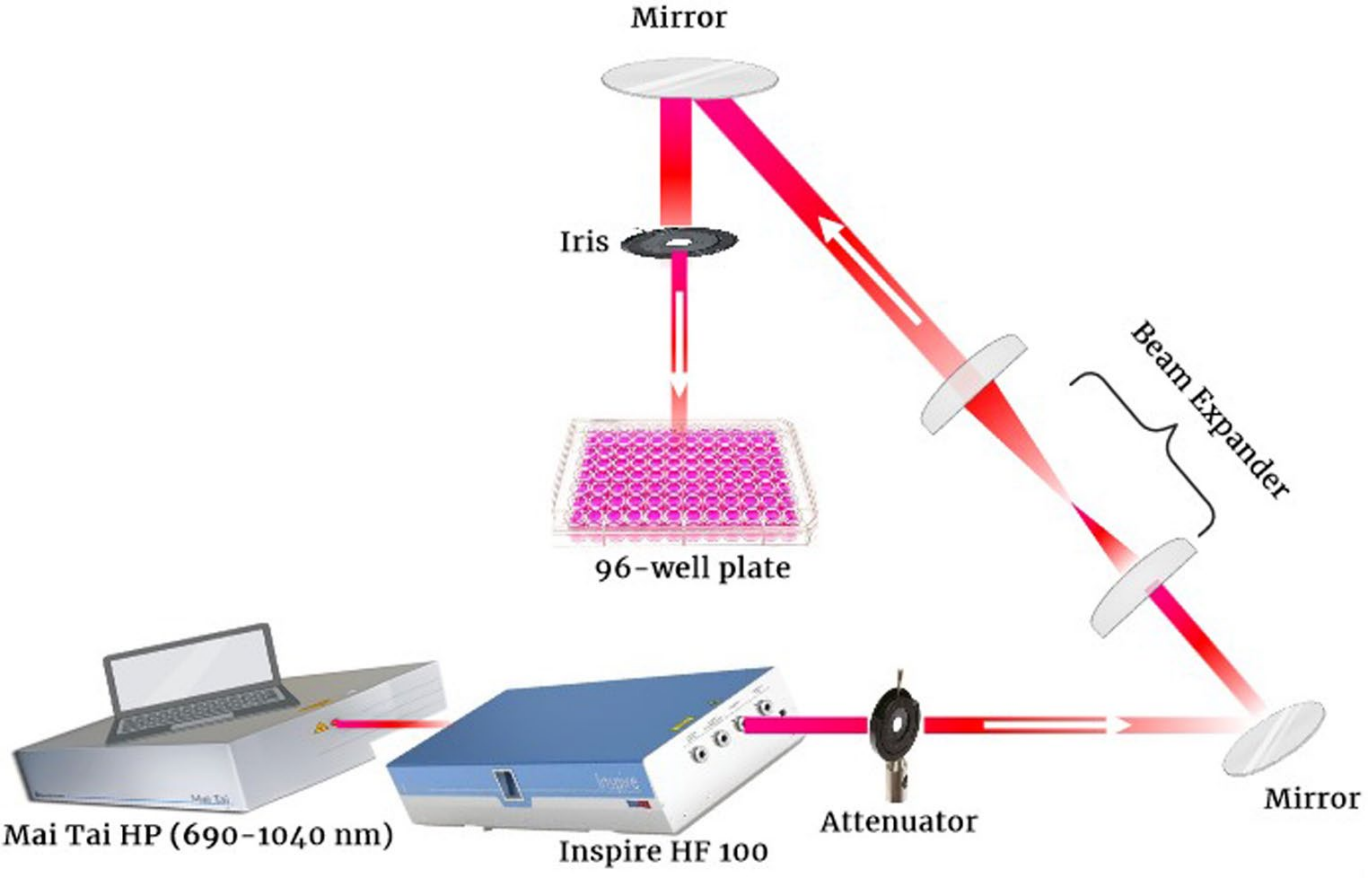
Diagram illustrating the experimental setup for exposing MDA-MB-231 and T47D cells to radiation

Photodynamic therapy was performed on human breast cancer cell lines using a 690 nm pulsed femtosecond laser (INSPIRE HF100, Spectra-Physics), targeting the Q-band absorption of TMPyP photosensitizer with a 200 mW mean power. The INSPIRE HF100 laser system (Spectra-Physics) was pumped by a mode-locked femtosecond Ti: sapphire MAI TAI HP laser (Spectra-Physics) to generate 690–1040 nm light pulses at an 80 MHz repetition rate and 1.5–2.9 W average power. As shown in Fig. 2, the 2 mm laser beam was expanded to 20 mm using a beam expander and directed toward the culture plate via mirrors. An iris diaphragm controlled the beam diameter. A laser power attenuator adjusted the beam power before it reached the cells, as measured by a Newport 843R power meter.

### Assessment of the toxicity of 690 nm laser irradiation, dark toxicity of TMPyP, and TMPyP-PDT on breast cancer cells

MDA-MB-231 and T47D cells (10^4^ cells/well) were seeded in 96-well plates and incubated at 37 °C with 5% CO_2_ for 24 h. Subsequently, cells were treated with fresh media containing increasing concentrations of TMPyP (2.5–70 µM). The laser plate medium was replaced with PS-free medium. After 24 h of incubation in the dark at 37 °C, all plates were refreshed with complete medium, except for the dark TMPyP plate, which was returned to the incubator. TMPyP-PDT and laser plates were irradiated with a 690 nm, 200 mW femtosecond pulsed laser for 10 min, followed by a 24-hour incubation period. Control cells were maintained without treatment or laser exposure.

Cell viability was assessed by adding 10 µL of 5 mg/mL MTT solution in PBS well and incubating at 37 °C for 3 h. Subsequently, the medium was replaced with 100 µL DMSO and mixed thoroughly to dissolve formazan crystals. Optical density (OD) at 570 nm was measured using a microplate reader (Thermo Fisher Scientific, USA). Results are presented as the mean ± SE of three independent experiments. The half-maximal inhibitory concentration (IC50) of TMPyP-mediated PDT, causing a 50% reduction in MDA-MB-231 and T47D cell viability, was determined by fitting dose-response curves to a non-linear regression equation using Prism 8 software (GraphPad Software Inc., San Diego, CA, USA).

### Evaluation of the chemotherapy and chemo-photodynamic combined therapy in vitro

A total of 10,000 cells of MDA-MB-231 and T47D were seeded per well in 96-well plates and incubated overnight. Cells were then treated with either DOX alone (0.0625–8 µg/mL) or a combination of DOX and TMPyP at its IC50 concentration for each cell line. After 24 h, the media was replaced with fresh growth media. Only cells treated with the DOX-TMPyP combination were irradiated with a 690 nm fs-laser (10 min, 200 mW). Cell viability was assessed 24 h later using the MTT assay.

### Detection of cellular ROS

To quantify reactive oxygen species (ROS) generation, cells were treated with 2’,7’-dichlorofluorescein diacetate (DCFH-DA). DCFH-DA is a cell-permeable, non-fluorescent compound cleaved by intracellular esterases to form the non-fluorescent DCFH. In the presence of ROS, DCFH is oxidized to highly fluorescent 2’,7’-dichlorofluorescein (DCF). Cells were seeded in 96-well plates at 10^4^ cells/well density and incubated for 24 h. The medium was then replaced with fresh medium containing either (1) no treatment (control & 690 nm laser), (2) DOX (0.0625 µg/ml), (3) TMPyP (IC50), or (4) a combination of DOX (0.0625 µg/ml) and TMPyP (IC50). After 24 h, the medium was replaced, and laser plates and plates containing TMPyP (IC50) alone or in combination with DOX were irradiated as described above and incubated for 3 h. Cells were washed with PBS and incubated with 100 µL of 5 µM H2DCFDA for 30 min at 37 °C in the dark. After two additional PBS washes, cells were imaged using a Leica DMi8 inverted microscope equipped with a Photometrics Prime 95B Scientific CMOS camera and a Leica EL6000 light source. Green fluorescence from DCF was captured using a FITC filter cube (excitation: 470/40 nm, emission: 520/40 nm).

### Flow cytometry analysis of ROS generation

The cells were inoculated at a density of 50,000 cells per well in 24-well plates. Following a 24-hour incubation, the medium was replaced with a new culture medium containing either (1) no treatment (control & 690 nm laser), (2) DOX (0.0625 µg/ml), (3) TMPyP (IC50), or (4) a combination of DOX (0.0625 µg/ml) and TMPyP (IC50). The medium was changed after 24 h, and laser plates, along with plates containing TMPyP (IC50) either alone or in conjunction with DOX were exposed to radiation as previously mentioned and incubated for three hours. Following a PBS wash, the cells were treated with DCFH-DA for 30 min at 37 °C in the dark. Subsequently, the cells were centrifuged, resuspended in PBS, and analyzed by BD FACS Calibur™ flow cytometer (BD Biosciences, USA) equipped with Cell Quest software.

### Apoptosis detection using flow cytometer

Cellular apoptosis was examined utilizing the Annexin V-fluorescein isothiocyanate (FITC)/propidium iodide (PI) dual staining method, which distinguishes the populations of live and apoptotic cells. MDA-MB-231 and T47D seeded in 24-well plates were treated with either (1) no drugs (control & 690 nm laser), (2) DOX (0.0625 µg/ml), (3) TMPyP (IC50), or (4) a combination of DOX (0.0625 µg/ml) and TMPyP (IC50). After the 24-hour incubation period, laser plates as well as plates containing TMPyP (IC50) either alone or in conjunction with DOX were exposed to a 690 nm fs-laser (10 min, 200 mW). Following a 24-hour incubation period, both floating and adhered cells were gathered through trypsinization and centrifuge at 1800 rpm for 5 min. The supernatants were poured off, and the cell pellets were rinsed twice with PBS. Subsequently, the cells were treated with Annexin V-FITC and PI according to the manufacturer’s instructions. In the end, apoptotic cell analysis was conducted by examining 10,000 gated cells with a BD FACS Calibur™ flow cytometer (BD Biosciences, USA) outfitted with Cell Quest software.

### RNA isolation and quantitative real-time polymerase chain reaction (RT-qPCR)

MDA-MB-231 and T47D cells were treated for 24 h with IC50 concentrations of TMPyP, DOX (0.0625 µg/mL), or their combination. Following medium replacement, cells treated with TMPyP (alone or with DOX) were exposed to 690 nm femtosecond laser light. Five experimental groups were used: control, DOX, laser alone, TMPyP + laser, and TMPyP + DOX + laser. Twenty-four hours post-irradiation, cells were harvested and lysed with TRIzol reagent (Thermo Fisher Scientific) according to the manufacturer’s protocol.

The Nanodrop was utilized to assess the concentration and purity of the extracted total RNA via measuring absorbance at A260 nm/A280 nm. The High-Capacity cDNA reverse transcription kit from Thermo Fisher Scientific, USA, was used to produce complementary DNA following the manufacturer’s directions. Eventually, the amplification of cDNA was performed via fluorescence-based relative quantitative PCR using SYBR Green qPCR Master Mix (Thermo Fisher Scientific, USA), to measure the relative mRNA levels of TGF-β, VEGFA, BCL-2, and BAX, standardized against GAPDH as a reference gene. The primers unique to each gene were developed by the IDT-PrimerQuest tool (http://eu.idtdna.com/primerquest/home/index) and confirmed using the IDT-OligoAnalyzer Tool (https://www.idtdna.com/calc/analyzer). The sequences of primers are shown in Table 1. The RT-PCR reaction was performed by mixing 12.5 µL of SYBR Green, 0.05 µL of ROX solution, 1 µL of each primer, and 2 µL of diluted cDNA template (1:5) in a final volume of 25 µL adjusted with DNase-free water. The amplification protocol was modified to include an initial denaturation phase of 10 min at 95 °C, followed by 40 cycles (denaturation at 94 °C for 15 s, annealing at 55 °C for 30 s, and extension at 72 °C for 30 s). To guarantee the purity and specificity of the amplified products, melting curves were established by rapidly heating the DNA to 95 °C for 15 s and then cooling to 60 °C. The mRNA was quantified using the comparative CT method (*n* = 2^–ΔΔCt^).

**Table 1:** The quantitative real-time PCR primer sequences

Gene	Forward primer (5’-3’)	Reverse primer (5’-3’)
TGF- β	TCCTGGCGATACCTCAGCAA	GTAGTGAACCCGTTGATGTCCA
VEGF-A	AGAAGGAGGAGGGCAGAAT	CACAGGATGGCTTGAAGATGTA
BCL-2	GTGGATGACTGAGTACCTGAAC	GAGACAGCCAGGAGAAATCAA
BAX	AGGTCTTTTTCCGAGTGGCA	GAAGTCCAATGTCCAGCCC
GAPDH	GTCGGAGTCAACGGATTTGGTC	GTTCTCAGCCTTGACGGTGC

### Statistical analysis

All experiments were conducted in triplicate, with three independent replicates per experiment. Data normality was assessed using the Shapiro-Wilk test. Data are presented as mean ± SEM. One-way ANOVA followed by Tukey’s post-hoc test was employed for multiple comparisons of normally distributed data. The student’s t-test was used to compare combined therapy and monotherapy. Statistical analysis was performed using SPSS version 20. Statistical significance was determined at a P-value of < 0.05.

## Results

### Optical studies and detection of singlet oxygen

**Fig. 3:**
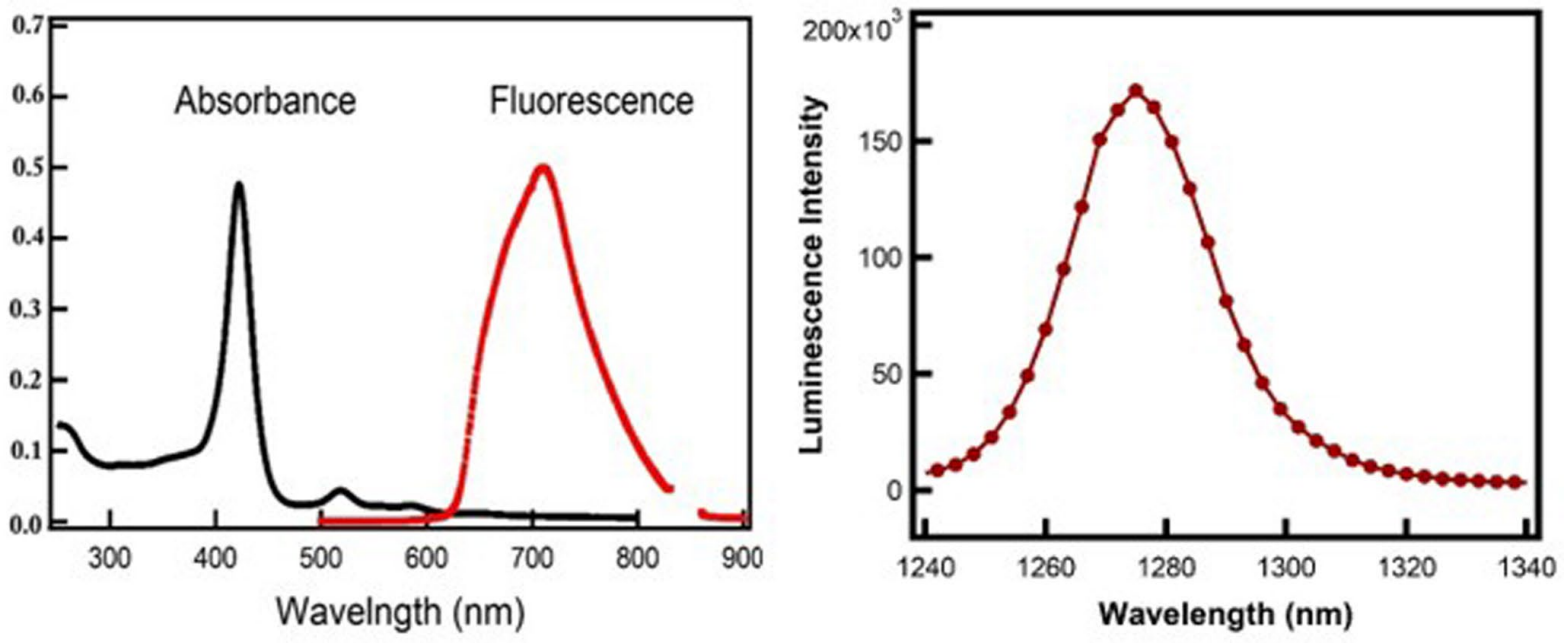
(Left) Absorption and fluorescence spectra of TMPyP in water. (Right) Luminescence spectra of the singlet oxygen produced for the investigated TMPyP

The UV-visible absorption spectrum of TMPyP in aqueous solution exhibited a characteristic Soret band centered at 422 nm, corresponding to the S_0_→S_2_ electronic transition, and weaker Q-bands at 518, 555, 585, and 650 nm, as depicted in Fig. 3A. The fluorescence emission spectrum of TMPyP, shown in the left panel of Fig. 3, displayed a broad band ranging from 600 to 800 nm, with a maximum emission wavelength at 710 nm. The potential of TMPyP to act as a photosensitizer for the photodynamic therapy of cancer is associated with its singlet oxygen quantum yield. The singlet oxygen quantum yield of TMPyP was determined to be 0.61, as shown in the right panel of Fig. 3, based on the relative intensities of the near-infrared emission bands of singlet oxygen centered at 1275 nm [25–27].

### The cytotoxic effects of TMPyP-PDT on MDA-MB-231 and T47D cells

**Fig. 4:**
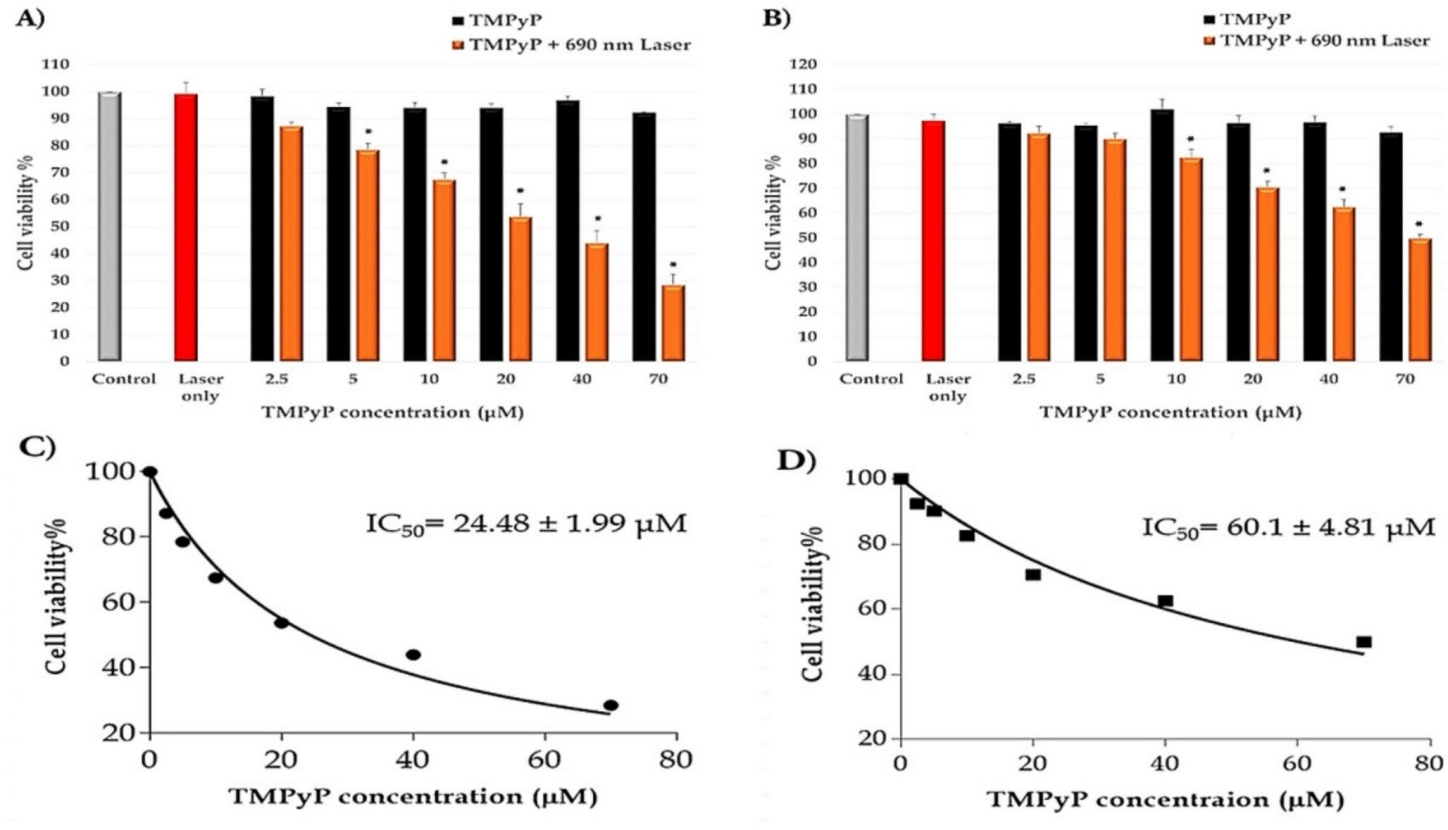
The cytotoxic effect of TMPyP, laser source, and TMPyP-mediated PDT. The cytotoxic effects of varying TMPyP concentrations were assessed on (**A**) MDA-MB-231 and (**B**) T47D cells, both with and without irradiation (dark toxicity). The cytotoxic effect of the laser exposure (200 mW, 10 min) in the absence of TMPyP was studied on both cell lines. Dose-response curves of (**C**) MDA-MB-231 and (**D**) T47D plotted from the results of MTT assays. The untreated cells were regarded as the control group. Data were subjected to one-way ANOVA followed by Tukeyʹs procedure at *p* < 0.05. Data show the means ± standard error of three separate experiments. *: significantly different (*P* < 0.05) compared to the control

The cytotoxic effects of TMPyP (2.5–70 µM) and laser irradiation were assessed individually on breast cancer cell lines. As shown in Fig. 4A and B, no significant dark toxicity of TMPyP or laser-induced cytotoxicity was observed, as cell viability remained above 90% (*P* > 0.05) in both cell lines. Conversely, our results demonstrated that TMPyP exhibited substantial phototoxicity against MDA-MB-231 and T47D cancer cells. TMPyP-PDT significantly reduced MDA-MB-231 cell viability at concentrations ranging from 5 µM (survival fraction: 78.75 ± 2.33%) to 70 µM (survival fraction: 28.53 ± 3.91%) of TMPyP (Fig. 4A). Similarly, TMPyP-PDT suppressed T47D cell growth at concentrations ranging from 10 µM (82.57 ± 3.33%) to 70 µM (49.88 ± 1.76%) of TMPyP (Fig. 4B). Notably, MDA-MB-231 cells displayed greater sensitivity to TMPyP-PDT compared to T47D, with IC50 values of 24.48 µM and 60.1 µM for MDA-MB-231 and T47D, respectively (Fig. 4C and D).

### The cytotoxic impacts of doxorubicin alone and in conjunction with TMPyP-PDT on breast cancer cell lines

**Fig. 5:**
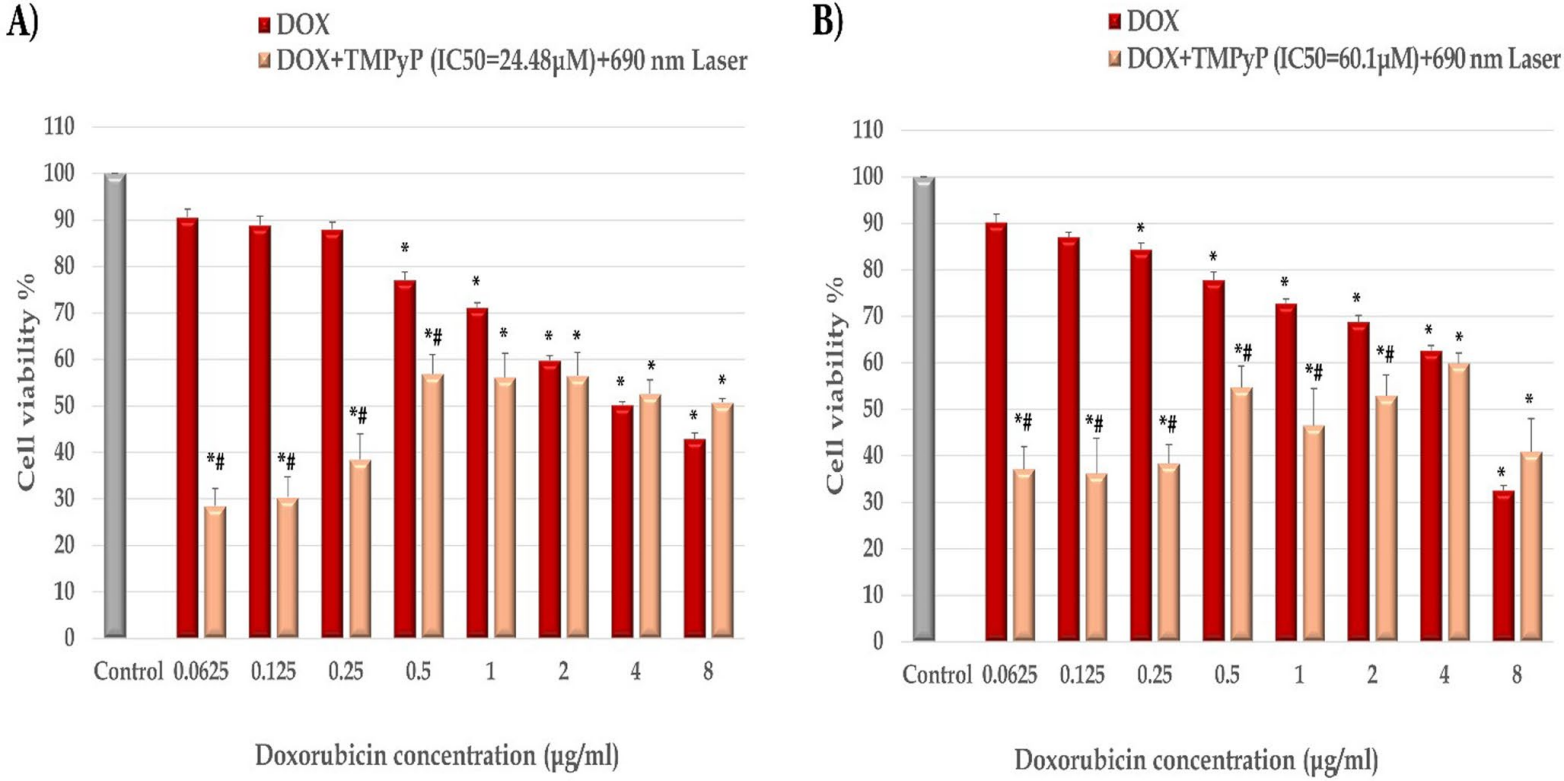
The cytotoxic effect of Doxorubicin monotherapy and in combination with TMPyP-PDT. The cytotoxic effect of different doses of DOX and the combination of these doses with IC50 values of TMPyP-PDT were studied on (**A**) MDA-MB-231 and (**B**) T47D cells. * and **#**: significantly different (*P* < 0.05) from the control and the corresponding DOX dose, respectively, by the student’s t-test

The anticancer effects of doxorubicin were assessed in MDA-MB-231 and T47D cells, as shown in Fig. 5A and B. A dose-dependent increase in cytotoxicity was observed with increasing doxorubicin concentrations (0.0625–8 µg/ml). The most significant anti-proliferative effects of doxorubicin were seen at concentrations ≥ 0.5 µg/ml in MDA-MB-231 and ≥ 0.25 µg/ml in T47D cells (*P* < 0.05). To determine the optimal combined concentration for combination therapy, the IC50 values of TMPyP-PDT for each cell line were paired with various doxorubicin concentrations. When combined with doxorubicin, TMPyP-PDT (IC50 = 24.48 µM) significantly enhanced cytotoxicity in MDA-MB-231 cells, particularly at lower doxorubicin doses (0.0625–0.5 µg/ml) compared to doxorubicin monotherapy (*P* < 0.001) (Fig. 5A). Similarly, T47D cell viability was significantly reduced when lower doses of doxorubicin (0.0625–2 µg/ml) were combined with the IC50 of TMPyP-PDT (60.1 µM) compared to doxorubicin alone (Fig. 5B). Increasing the doxorubicin concentration in combination therapy reduced the significant difference between monotherapy and combined therapy in both breast cancer cell lines.

### Production of intracellular reactive oxygen species

**Fig. 6:**
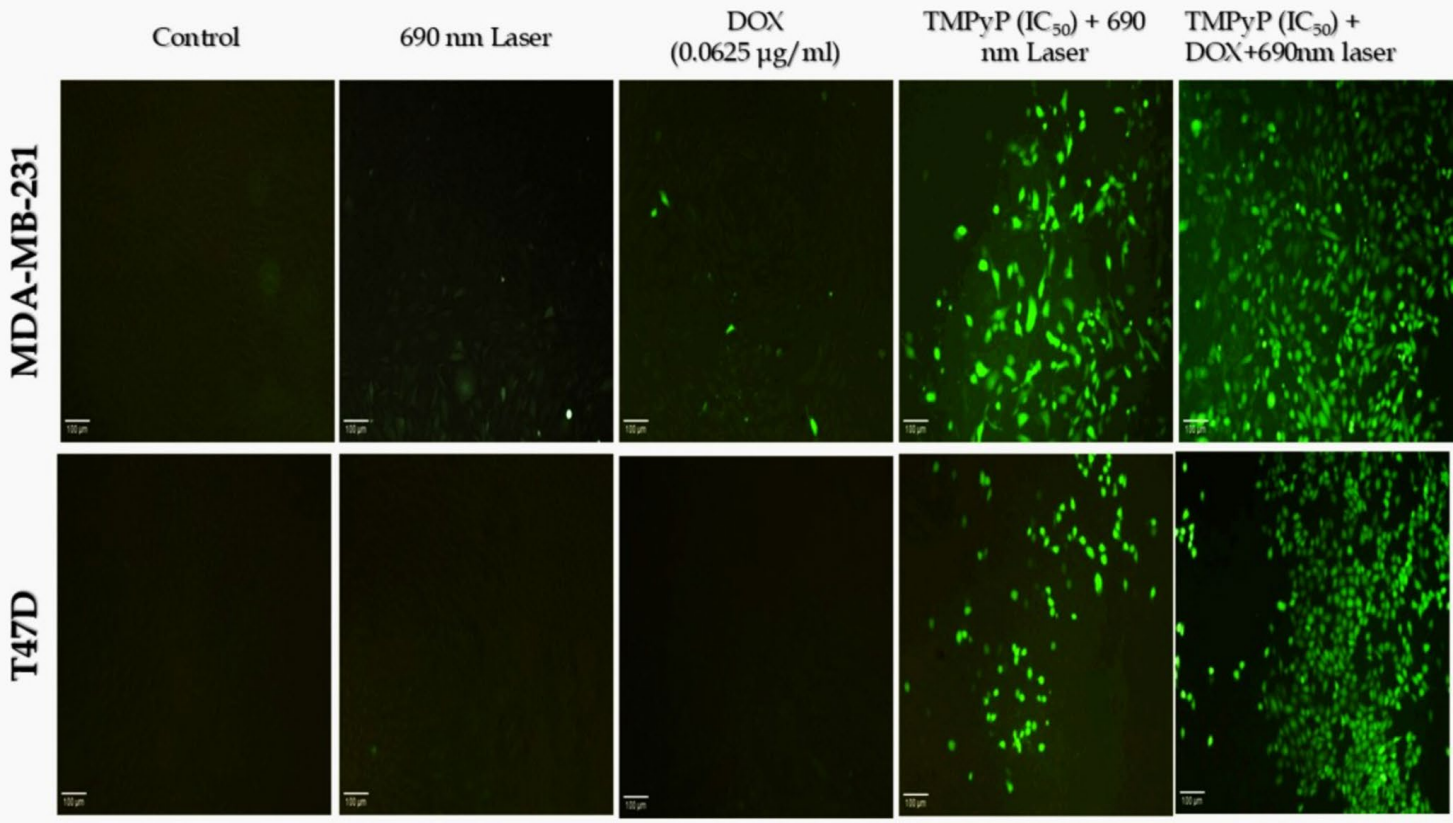
Fluorescence microscope pictures showing green DCFH-DA staining for intracellular ROS production in MDA-MB-231 and T47D breast cancer cell lines. Bar = 100 *µ*m

The cancer-fighting impact of a photosensitizer depends on the cell death and adaptive reactions driven by ROS. Consequently, we explored the cellular ROS stress induced by chemotherapy, photodynamic therapy, and their combination using the DCFH-DA probe. As illustrated in Fig. 6, Confocal microscopy employing DCFH-DA revealed enhanced green fluorescence, suggesting heightened ROS stress, particularly in the TMPyP-PDT and DOX + TMPyP-PDT groups for both MDA-MB-231 and T47D when compared to the untreated control. Groups subjected to laser treatment alone and a low dose of DOX (0.0625 µg/ml) did not show notable DCFH-DA fluorescence when compared to untreated controls in both cell lines.

**Fig. 7:**
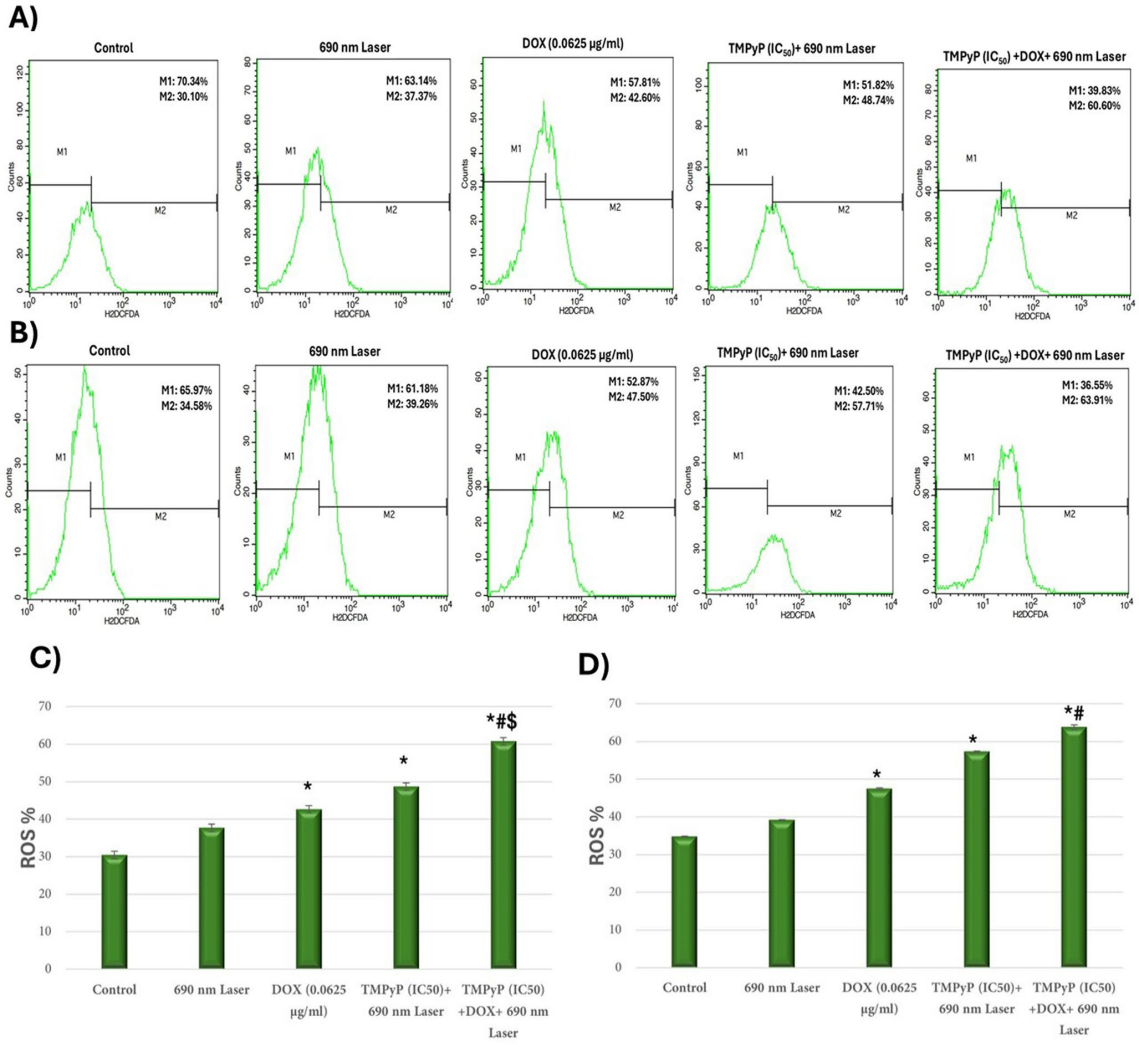
Flow cytometric analysis of intracellular ROS within each group of **(A)** MDA-MB-231 and **(B)** T47D cells utilizing the DCFH-DA probe. Histograms **(C)** and **(D)** depict the quantitative assessment of the percentage of ROS+ (M2) cells in the groups of MDA-MB-231 and T47D cells, respectively. ^(*)^ Significance versus control, ^(#)^ Significance against doxorubicin monotherapy, ^($)^ Significance against TMPyP-PDT monotherapy

The quantification analysis of intracellular ROS was further validated through flow cytometric evaluation using the same probe for MDA-MB-231 and T47D (Fig. 7A and B, respectively). The intracellular ROS levels significantly increased in the DOX (0.0625 µg/ml), TMPyP-PDT, and DOX + TMPyP-PDT groups relative to the untreated control for both MDA-MB-231 and T47D cells. Notably, the group receiving combined treatment (DOX + TMPyP-PDT) for MDA-MB-231 and T47D showed the highest ratios of ROS + cells, achieving 60.7% and 63.7%, respectively (Figs. 7C & D). Furthermore, this ROS increase in the DOX + TMPyP-PDT group is significant when contrasted with the DOX (0.0625 µg/ml) and TMPyP-PDT monotherapy groups in MDA-MB-231 cells (Fig. 7C) and with the DOX (0.0625 µg/ml) group alone in T47D (Fig. 7D).

### Staining with Annexin V/PI for apoptosis analysis

**Fig. 8:**
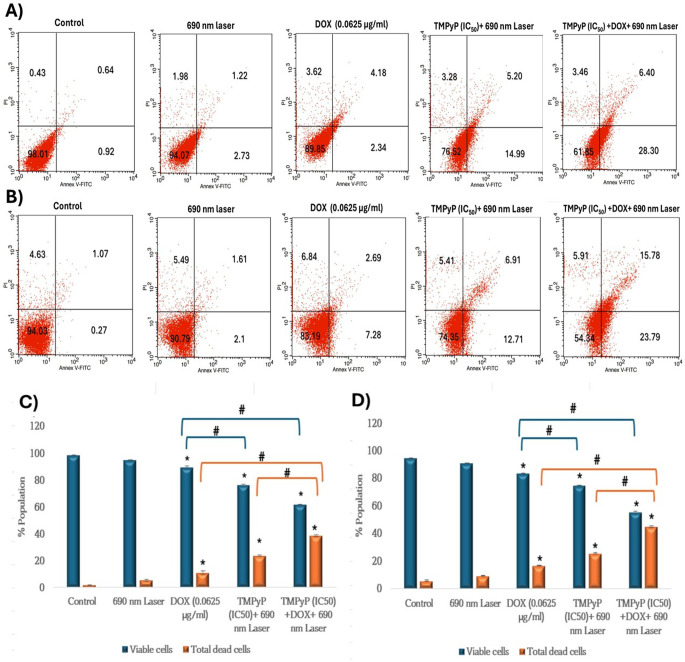
Influence of chemotherapy, photodynamic therapy & their combination on apoptosis. **(A) & (B)** Dot plots of Annexin V/PI flow cytometric analysis of MDA-MB-231 and T47D treated cells, respectively. Untreated cells were used as a control. Numbers represent the % population in each quadrant. (**C) & (D)** percentage of the viable and total dead cell populations as determined by flow cytometry for MDA-MB-231 and T47D, respectively. Data were represented as mean ± SD of three independent experiments. The symbol (*) represents significance relative to the control, while the symbol (#) represents significance with the indicated groups

To assess the therapeutic efficacy of photodynamic therapy and its combination with a low dose of dox for inducing apoptotic cell death, MDA-MB-231, and T47D cells received treatment with low dose DOX (0.0625 µg/ml), TMPyP-PDT (IC50), and their combination. The data presented in Fig. 8 (A—D) indicated that the monotherapies using either DOX or TMPyP-PDT and their combination demonstrate a significant rise in the dead cell population relative to control in MDA-MB-231 and T47D (*P* < 0.05). Interestingly, the combination treatment of TMPyP-PDT and DOX resulted in a significantly greater number of dead cells, reaching 38.5% (*P* < 0.05), when compared to the DOX (10.90%) and TMPyP-PDT (23.67%) groups of MDA-MB-231 cells (Fig. 8C). Furthermore, the combined treatment of TMPyP-PDT and DOX demonstrated a significantly increased number of dead cells, reaching 44.76% (*P* < 0.05) compared to the DOX (16.62%) and TMPyP-PDT (25.52%) groups of T47D cells (Fig. 8D).

### The effect of DOX and TMPyP-PDT and their combination on the angiogenesis and apoptosis of breast cancer cell lines

Quantitative RT-PCR was used to analyze mRNA expression of the angiogenesis regulators, transforming growth factor-β (TGF-β) and vascular endothelial growth factor A (VEGF-A).

**Fig. 9:**
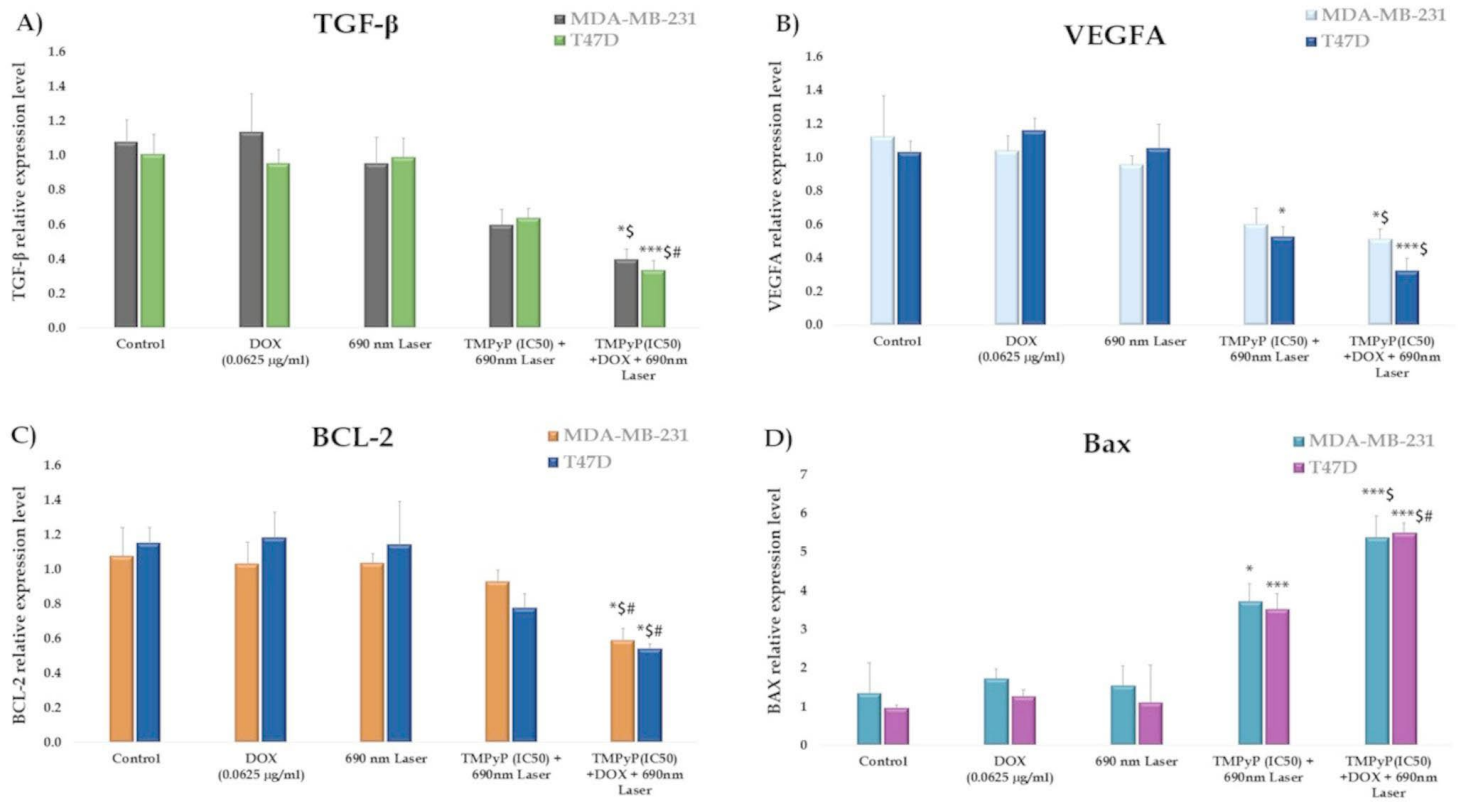
The influence of photodynamic treatment, chemotherapy, and the combination thereof on the mRNA levels of the angiogenesis-related genes (TGF-β and VEGF-A**)** and apoptosis-related genes (BCL-2 and BAX) in MDA-MB-231 and T47D cells. Using the One-Way ANOVA test, the means ± SEM are shown by bars. The student’s T-test was used to determine any significant differences between the two groups. Results are significant at *P* ≤ 0.05 and extremely significant at *P* ≤ 0.001. ^(*)^ Significance versus control, ^($)^ Significance against doxorubicin monotherapy, ^(#)^ Significance against TMPyP-PDT monotherapy

- Combined low-dose doxorubicin (DOX, 0.0625 µg/ml) treatment significantly reduced TGF-β mRNA expression in MDA-MB-231 (*P* < 0.05) and T47D (*P* < 0.001) cells compared to untreated controls (Fig. 9A). This reduction was also significant relative to DOX monotherapy in MDA-MB-231 cells (*P* < 0.05) and to both DOX and TMPyP-PDT monotherapies in T47D cells (*P* < 0.05).
- VEGF-A mRNA expression was significantly decreased in MDA-MB-231 cells following combined treatment (*P* < 0.05) and in T47D cells after TMPyP-PDT (*P* < 0.05) and combined treatment (*P* < 0.001) compared to controls (Fig. 9B). The combined therapy also significantly lowered VEGF-A mRNA levels compared to DOX monotherapy in both cell lines (*P* < 0.05).

The relative mRNA expression of apoptosis-related genes BCL-2 and BAX was compared in MDA-MB-231 and T47D cells. Figure 9C revealed a significant decrease in BCL-2 expression in both cell lines following combined therapy compared to control, DOX, and TMPyP-PDT treatments (*P* < 0.05). Neither DOX nor TMPyP-PDT monotherapy significantly altered BCL-2 expression relative to the control. In contrast, BAX expression significantly increased in both cell lines after TMPyP-PDT (*P* < 0.05) and combined therapy (*P* < 0.001). Furthermore, combined therapy significantly elevated BAX expression in MDA-MB-231 cells compared to DOX alone and in T47D cells compared to both DOX and TMPyP-PDT alone.

## Discussion

Doxorubicin (DOX) remains a widely studied chemotherapeutic agent due to its efficacy against various cancers. However, its clinical use is limited by cardiotoxicity and the development of drug resistance [28, 29]. Photodynamic therapy (PDT) is an emerging therapeutic modality with potential advantages over traditional therapies, including a reduced risk of drug resistance [30]. Recent research suggests that combining PDT with chemotherapy can enhance therapeutic outcomes by synergistically targeting cancer cells, potentially leading to improved efficacy and reduced side effects [31, 32].

We investigated the efficacy of photodynamic therapy (PDT) using the cationic porphyrin photosensitizer TMPyP and femtosecond laser irradiation at the Q band (690 nm) to exploit the therapeutic window (600–1200 nm) for deeper tissue penetration [33]. TMPyP-PDT demonstrated dose-dependent inhibition of breast cancer cell lines MDA-MB-231 and T47D. MDA-MB-231 cells displayed higher sensitivity to TMPyP-PDT (IC50 = 24.48 µM) compared to T47D cells (IC50 = 60.1 µM). These results align with previous studies reporting the effectiveness of TMPyP-PDT against human ovarian carcinoma A2780 and hepatocellular carcinoma HePG2 cells [34, 35]. Additionally, TMPyP-PDT significantly reduced the viability of HeLa cervical cancer cells and skin malignant melanoma G361 cells to 3.8% and 9.4%, respectively, at a TMPyP concentration of 5 µM and blue light irradiation at 414 nm [36]. Our findings indicate that neither TMPyP (up to 70 µM) nor femtosecond pulsed laser irradiation alone affected the growth of MDA-MB-231 and T47D cells. However, a previous study reported significant dark cytotoxicity of TMPyP against A2780 ovarian cells at concentrations ranging from 3 to 60 µM [34].

This study investigated the cytotoxic effects of low-dose doxorubicin (DOX), both alone and in combination with TMPyP-mediated photodynamic therapy (PDT), on MDA-MB-231 and T47D breast cancer cells. Consistent with prior research demonstrating enhanced efficacy through combined PDT and chemotherapy, we hypothesized a synergistic effect. Previous studies have shown that combining PDT with DOX or other photosensitizers like ZnPc, PhA, and ALA, significantly inhibits cancer cell proliferation and reduces cell viability in various cell lines, including MCF-7, HeLa, and leukemic murine cells [37–39]. Moreover, the timing of DOX administration relative to PDT appears critical, with simultaneous administration showing greater potentiation of photodynamic effects [40].

Consistent with prior research, DOX displayed concentration-dependent cytotoxicity in MDA-MB-231 and T47D cells. Combining DOX with TMPyP-PDT significantly increased DOX cytotoxicity, with the maximum effect observed at 0.0625 µg/ml DOX, reducing cell viability to 28.55% (MDA-MB-231) and 37.12% (T47D). At this concentration, DOX alone showed minimal impact. The combination’s efficacy diminished with increasing DOX concentration. This observation aligns with previous reports of ground-state interactions between DOX and TMPyP, causing reduced TMPyP absorption and a red shift in its Soret band [35]. Similar antagonistic effects have been reported for HPD-PDT and Adriamycin combinations [41].

ROS production is the primary mechanism underlying tumor cell death induced by PDT [42]. To investigate the impact of chemotherapy, PDT, and their combination on intracellular ROS levels, we assessed these levels in MDA-MB-231 and T47D cells. Our microscopic analysis showed that MDA-MB-231 and MCF7 cancer cells treated simultaneously with low-dose DOX (0.0625 µg/ml) and TMPyP-PDT had a markedly greater rise in ROS levels than those treated with either TMPyP-PDT or DOX, which showed a moderate ROS production for TMPyP-PDT and a minimal level for DOX. These findings were validated through quantitative analysis of ROS-positive cells via flow cytometry, which demonstrated that the combination of TMPyP-PDT and DOX in chemo-photodynamic therapy can increase ROS production compared to each therapy used individually. Consistent with our findings, research indicated that intracellular ROS content was significantly elevated by combining DOX–CUR–PFOB–PLGA NPs with PDT [43]. Furthermore, it was observed that both DOX and PDT Me-ALA treatments resulted in significantly elevated levels of ROS in glioblastoma (GBM) cell lines [44]. The results indicate that the combination of chemotherapy and photodynamic therapy markedly increases total ROS production compared to the separate impacts of each therapy, offering a hopeful direction for enhancing cancer treatment effectiveness.

Elevated levels of the inflammatory cytokine transforming growth factor-beta (TGF-β) in breast tumors are associated with advanced disease stages and poor prognosis, often leading to bone and lung metastases [45]. Studies have shown that increased TGF-β1 and vascular endothelial growth factor-A (VEGF-A) promote angiogenesis and extracellular matrix formation around tumor cells [46]. Previous research has demonstrated that TGF-β significantly upregulates both VEGF mRNA and protein expression [47, 48]. Our findings suggest that the combined chemo-photodynamic therapy of low-dose DOX and TMPyP-PDT induces cytotoxic effects, including the suppression of TGF-β and VEGF-A cytokine expression. Our results indicate that the combined therapy effectively suppressed TGF-β expression in MDA-MB-231 (*P* < 0.05) and T47D (*P* < 0.001) cells compared to the control. However, neither DOX nor TMPyP-PDT alone significantly reduced TGF-β mRNA levels in either cell type. Similarly, in MDA-MB-231 cells, only the combined therapy decreased VEGF-A mRNA levels compared to the control. In T47D cells, both TMPyP-PDT alone and the combined therapy significantly lowered VEGF-A mRNA levels (*P* < 0.05, *P* < 0.001, respectively). Therefore, the combination of DOX and TMPyP-PDT in chemo-photodynamic therapy is more effective in reducing TGF-β and VEGF-A expression than either treatment alone.

To clarify the impact of DOX, TMPyP-PDT, and their combination on the apoptosis of MDA-MB-231 and T47D cells, the total apoptotic cell population for each treatment was assessed using Annexin V staining and subsequently analyzed via flow cytometry. The findings validated the success of the combined treatment by markedly increasing the apoptotic cell population to 38.5% for MDA-MB-231, compared to monotherapy with DOX (10.90%) and TMPyP-PDT (23.67%). Furthermore, the highest rate of apoptosis stimulation in T47D cells was observed in the combined therapy group (44.76%) in comparison to DOX (16.62%) and TMPyP-PDT (25.52%) treatments. The increase in apoptosis observed in the combination therapy group indicates that the concurrent activation of various cell death pathways (stemming from both chemotherapeutic and photodynamic influences) results in enhanced cancer cell destruction. Our results are consistent with a prior study that demonstrated a significant increase (~ 36%) in the apoptosis rate in the nanoparticle-encapsulated DOX and curcumin group of MCF cells following irradiation treatment, compared to DOX alone (~ 4%) [49]. Additionally, in line with previous research, the highest apoptosis induction rate in MDA-MB-231 cells was observed with the combined treatment of tamoxifen and ZnPc-PDT [50].

To elucidate the molecular mechanisms underlying the anticancer effects of DOX, TMPyP-PDT, and their combination on MDA-MB-231 and T47D cells, we assessed the expression levels of the apoptosis-related genes BCL-2 and BAX. Our results indicated that BCL-2 expression was significantly downregulated only in the dual treatment group of MDA-MB-231 cells compared to the control, low-dose DOX, and TMPyP-PDT monotherapy groups. Conversely, the pro-apoptotic BAX protein was significantly upregulated in both the TMPyP-PDT and combined therapy groups of MDA-MB-231 and T47D cells compared to the control group. These findings are consistent with previous reports by Zhang et al. [49], who demonstrated that nanoparticle-encapsulated DOX and Curcumin, upon photochemical activation, induced apoptosis in MCF-7 cells by downregulating BCL-2 and upregulating BAX. Similarly, another study [50] showed that ZnPc-PDT combined with Tamoxifen enhanced apoptosis by suppressing Bcl-2 expression.

## Conclusion

The study demonstrates that monotherapies with DOX and TMPyP-PDT significantly increase cytotoxicity against MDA-MB-231 and T47D breast cancer cells in a concentration-dependent manner. Our findings indicate that combining TMPyP-PDT and DOX significantly enhances DOX sensitivity, particularly at lower DOX concentrations. Furthermore, co-treatment of MDA-MB-231 and T47D cells with TMPyP-PDT and low-dose DOX significantly increases reactive oxygen species (ROS) production compared to the control and monotherapy groups. Additionally, the combined treatment effectively inhibits angiogenesis by downregulating TGF-β and VEGF-A cytokines. Moreover, the combination of low-dose DOX and TMPyP-PDT enhances the apoptotic rate of MDA-MB-231 and T47D cells due to increased ROS production. The combined therapy also promotes apoptosis by decreasing Bcl-2 expression while increasing BAX expression in MDA-MB-231 and T47D cells compared to single treatments with DOX or TMPyP-PDT. These findings suggest a combination effect of the dual therapy, potentially minimizing chemotherapy-associated toxicity and resistance. However, further clinical trials are necessary to translate these results into clinical practice.

## Data Availability

No datasets were generated or analysed during the current study.
